# Dermatofibrosarcoma protuberans mimicking hemorrhoids: a rare case report

**DOI:** 10.1097/RC9.0000000000000615

**Published:** 2026-07-08

**Authors:** Sofyan Abu Freih, Elchanan Quint, Anton Osyntsov, Ismaell Massalha, Ilia Pinsk

**Affiliations:** a Department of General Surgery, Soroka University Medical Center, Beer-Sheva, Israel; b Department of Oncology, Soroka University Medical Center, Beer-Sheva, Israel

**Keywords:** dermatofibrosarcoma protuberans, hemorrhoids, perianal neoplasms, sarcoma, soft tissue neoplasms

## Abstract

**Introduction and importance:**

Dermatofibrosarcoma protuberans is a rare soft tissue sarcoma. Perianal involvement is exceptional and may mimic benign anorectal disease, leading to delayed diagnosis.

**Case presentation:**

A 28-year-old man presented with 6 months of anal pain, pruritus, and intermittent bleeding, initially treated as hemorrhoids. Surgical excision of presumed hemorrhoidal tissue revealed dermatofibrosarcoma protuberans with fibrosarcomatous transformation and focally involved margins. Molecular testing confirmed COL1A1-PDGFB fusion. Staging PET-CT showed localized disease without distant metastasis. After multidisciplinary discussion, the patient underwent radical re-excision, including part of the internal anal sphincter. No residual malignancy was identified, and final margins were clear. Follow-up clinical assessment and imaging showed no evidence of recurrence.

**Clinical discussion:**

Perianal dermatofibrosarcoma protuberans is rare and can resemble common anorectal conditions. Fibrosarcomatous transformation increases the risk of recurrence and requires complete excision with negative margins. Histopathology and molecular testing are essential for diagnosis. Long-term surveillance is required because recurrence may occur years after treatment.

**Conclusion:**

Persistent or atypical anorectal symptoms should prompt a biopsy. Complete excision with negative margins remains the key treatment principle.

## Introduction

Dermatofibrosarcoma protuberans (DFSP) is a rare, locally aggressive soft tissue sarcoma, accounting for less than 0.1% of all malignancies and approximately 1% of soft tissue sarcomas^[^1,2^]^. It usually arises on the trunk and extremities, while perianal involvement is exceptionally uncommon^[^2–4^]^. Rare cases have also been reported in other unusual anatomical locations, including the orbit and vulva^[^5,6^]^. DFSP is characterized by slow infiltrative growth and a high risk of local recurrence when excision is incomplete^[^2,7,8^]^.

Diagnosis may be challenging because DFSP often resembles benign dermatologic or soft tissue conditions. In the perianal region, symptoms such as pain, pruritus, and bleeding are frequently attributed to common anorectal diseases, including hemorrhoids or fissures, resulting in delayed diagnosis^[^3,9^]^. Histopathology, supported by immunohistochemistry and molecular testing, particularly the detection of the COL1A1-PDGFB fusion gene, is important for definitive diagnosis^[^1,4^]^.

HIGHLIGHTS

- Patients with persistent anorectal symptoms that do not respond to standard treatment should undergo a biopsy to exclude rare malignancies.
- DFSP may closely mimic hemorrhoids and delay diagnosis, making early tissue assessment important.
- Molecular confirmation of the COL1A1-PDGFB fusion improves diagnostic accuracy.
- Achieving negative surgical margins is essential to prevent recurrence.
- Long-term surveillance is required because late recurrence can occur even after complete excision.

Complete surgical excision with negative margins remains the cornerstone of treatment^[^8,10^]^. Fibrosarcomatous transformation represents a more aggressive variant associated with increased recurrence risk and may require wider excision and closer surveillance^[^4,7^]^. We report a rare case of perianal DFSP with fibrosarcomatous transformation, initially misdiagnosed as hemorrhoids.

### Discussion of differential diagnosis

At presentation, working diagnoses included thrombosed hemorrhoids, chronic anal fissure, perianal abscess, hidradenitis suppurativa, and soft tissue tumors such as liposarcoma. DFSP was not initially suspected because of its rarity and its similarity to benign anorectal disease. Persistent symptoms and atypical intraoperative appearance prompted reevaluation. Histopathology and molecular fusion testing ultimately supported the diagnosis^[^1,4^]^.

## Case presentation

Timelines of clinical course are the following:

- Six months before the presentation
  - Anal pain, pruritus, and intermittent bleeding
  - Diagnosed clinically as hemorrhoids
  - Conservative treatment with no improvement
- May 2023
  - Surgical excision of presumed hemorrhoids
- Postoperative pathology
  - Malignant mesenchymal tumor ? DFSP with fibrosarcomatous transformation
- November 2023
  - PET-CT: Localized uptake, no metastasis
- December 2023
  - Radical re-excision with partial internal sphincter
- Mid-2024
  - Colonoscopy normal
- Later 2024
  - Axillary lymph node ? reactive on PET-CT
- Final surgery
  - Re-excision with clear margins
- Follow-up
  - No recurrence

A 28-year-old man presented with a 6-month history of anal pain, pruritus, and intermittent rectal bleeding. He had initially been diagnosed with hemorrhoids and received conservative treatment without improvement. He had no significant medical or family history but reported smoking one pack of cigarettes daily for 6 years.

**Figure 1.**
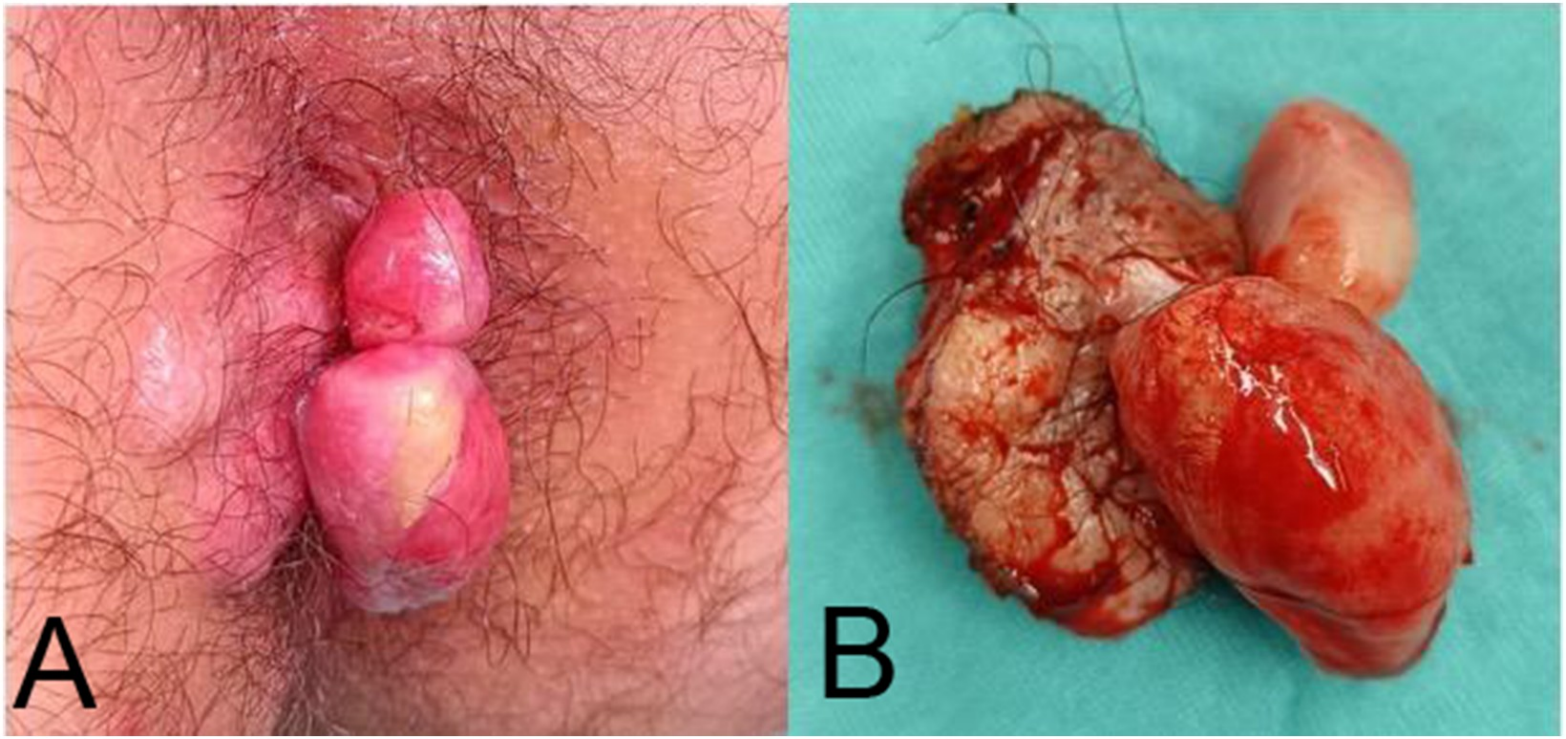
Clinical and intraoperative findings. (A) Prolapsed perianal lesion initially diagnosed as hemorrhoids. (B) Surgical specimen showing excised lesion with underlying perianal mass.

Physical examination demonstrated a prolapsed perianal lesion with two adjacent subcutaneous masses (Fig. 1A). The clinical findings were considered consistent with advanced hemorrhoidal disease, and the patient underwent surgical excision in May 2023 (Fig. 1B). No preoperative biopsy was performed because the lesion appeared compatible with hemorrhoids.

**Figure 2.**
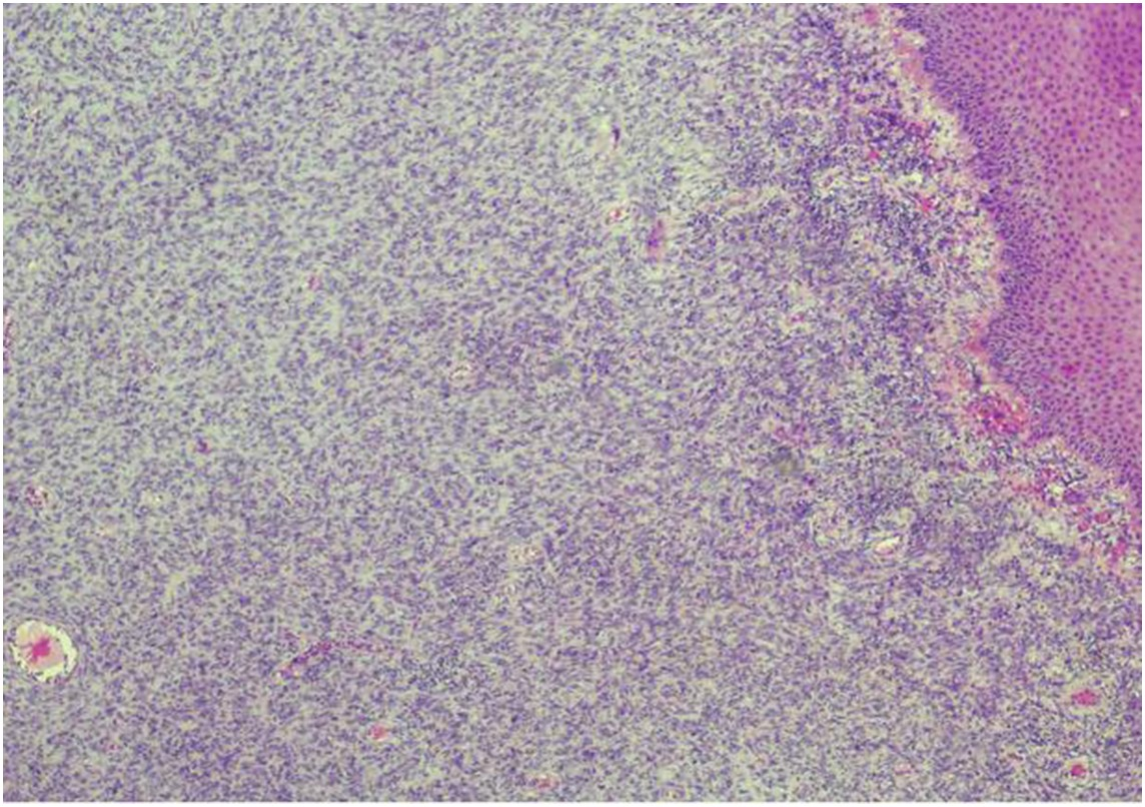
Histopathological findings of the excised lesion.

**Figure 3.**
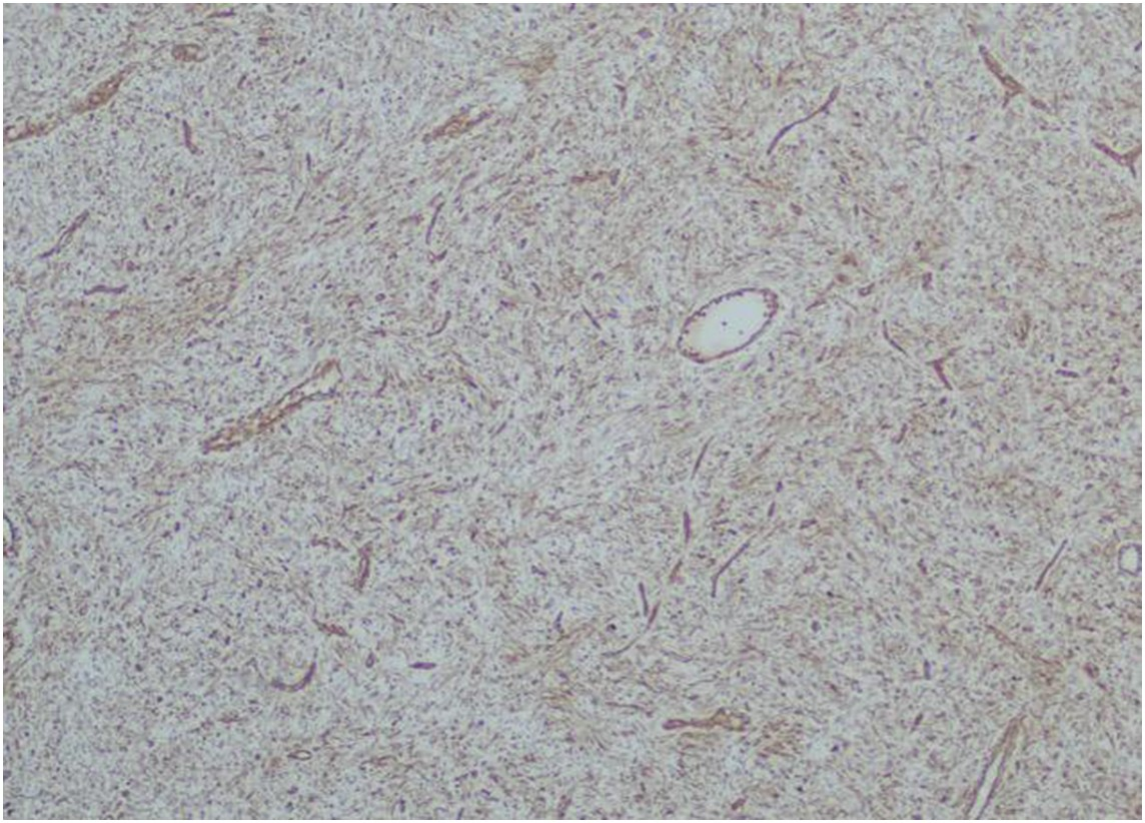
Immunohistochemical findings.

Histopathological examination demonstrated a malignant spindle-cell neoplasm with fibrosarcomatous transformation, atypical mitoses, and focal myxoid changes. Immunohistochemistry supported the diagnosis, and molecular testing confirmed COL1A1-PDGFB fusion consistent with dermatofibrosarcoma protuberans (Figs 2 and 3). Surgical margins were focally involved.

Spindle cell proliferation arranged in a storiform pattern consistent with dermatofibrosarcoma protuberans.

Tumor cells showing immunohistochemical staining pattern supporting the diagnosis of dermatofibrosarcoma protuberans.

PET-CT demonstrated localized uptake in the perianal region without distant metastasis. Following multidisciplinary discussion, radical re-excision was recommended because of positive margins and fibrosarcomatous transformation. In December 2023, the patient underwent radical re-excision, including partial internal anal sphincter resection (Supplemental Digital Content Figure 1, available at: http://links.lww.com/IJSCR/A76). Histopathological analysis demonstrated no residual malignancy and clear margins.

Follow-up clinical examinations and imaging showed no evidence of recurrence. Colonoscopy performed during follow-up was unremarkable. The patient remains asymptomatic under ongoing surveillance.

This case report has been presented in line with the SCARE 2025 criteria^[^11^]^.

## Discussion

Perianal dermatofibrosarcoma protuberans is exceptionally rare and may mimic common benign anorectal conditions, resulting in delayed diagnosis. In the present case, the patient’s symptoms and clinical findings were initially attributed to hemorrhoids, which postponed definitive diagnosis until histopathological examination following surgical excision. Similar diagnostic delays have been reported in previous cases of perianal DFSP^[^3,9^]^.

Definitive diagnosis relies on histopathological evaluation supported by immunohistochemistry and molecular testing. Detection of the COL1A1-PDGFB fusion gene is highly characteristic and supports diagnostic confirmation in challenging cases^[^1,4^]^. Fibrosarcomatous transformation represents a more aggressive DFSP subtype associated with higher rates of local recurrence and metastatic potential^[^4,7^]^.

Complete surgical excision with negative margins remains the cornerstone of treatment^[^8,10,12^]^. Inadequate initial excision significantly increases recurrence risk and may necessitate repeat surgery^[^7,8,12^]^. In our patient, positive margins after the initial operation required radical re-excision to achieve complete tumor clearance. Preservation of anal function while ensuring oncologic control represented an important surgical consideration.

Long-term surveillance is essential because recurrence may occur years after treatment, particularly in patients with fibrosarcomatous transformation^[^4,7,13^]^. This case highlights the importance of considering uncommon malignancies in patients with persistent or atypical anorectal symptoms and supports early biopsy when clinical findings are unusual or refractory to standard treatment.

## Conclusion

Perianal dermatofibrosarcoma protuberans is a rare malignancy that may clinically mimic hemorrhoids and delay diagnosis. Persistent or atypical anorectal symptoms should prompt further evaluation and consideration of biopsy. Complete surgical excision with negative margins remains essential for local disease control, particularly in cases with fibrosarcomatous transformation^[^8,10,12^]^. Long-term follow-up is required because of the risk of late recurrence.

## Data Availability

All data supporting this case are included within the manuscript.
